# FluoTag-EMSA: a fast and accessible quantitative method to assess RNA-binding specificity using 3′-tagged hybrid duplexes

**DOI:** 10.3389/fmolb.2025.1727371

**Published:** 2025-12-10

**Authors:** Benjamin Rothé, Daniel B. Constam

**Affiliations:** Ecole Polytechnique Fédérale de Lausanne (EPFL) SV ISREC, Lausanne, Switzerland

**Keywords:** RNA-protein interactions, RNA-binding protein, electrophoretic mobility shift assay (EMSA), non-radioactive detection, fluorescent RNA labeling, RNA tagging, FluoTag-EMSA, ribonucleoprotein complexes (RNPs)

## Abstract

Electrophoretic mobility shift assay (EMSA) is widely used to study RNA-protein interactions but remains limited by safety, cost, and time constraints associated with radioactive or covalent fluorescent labeling. Here, a novel method termed FluoTag-EMSA overcomes these hurdles by providing the RNA 3′ends with short sequence tags that can be hybridized to specific complementary fluorescent DNA probes, eliminating the need for chemical or enzymatic labeling steps. Specifically, two independent sequence tags are described that do not disrupt RNA folding and allow efficient annealing to complementary oligonucleotides carrying far-red/near-infrared dyes (700 nm and 800 nm), enabling direct in-gel fluorescence detection. Both tag/probe duplexes exhibit identical thermodynamic properties and can be used interchangeably. FluoTag-EMSA is a streamlined and reproducible non-radioactive alternative method for studying RNA-protein interactions without the need for specialized equipment.

## 1 Introduction

RNA-binding proteins (RBPs) govern numerous processes of post-transcriptional gene regulation, including mRNA splicing, translation, and decay. Electrophoretic mobility shift assay (EMSA) is a powerful tool and widely used to detect and quantify protein-RNA interactions involved in these processes and their regulation (Garner and Revzin, 1981). However, conventional EMSA typically relies on radiolabeled probes that present safety, regulatory, and workflow constraints. Several fluorescence-based EMSA protocols have also been described but typically require the individual fluorescent labeling of each RNA of interest through time-consuming in-house conjugation or expensive custom synthesis, thereby severely limiting throughput and flexibility (Pagano et al., 2011; Stowell et al., 2021).

Recently, we developed an alternative RNA labeling strategy by providing the template of the RNA synthesis reaction with a 21 nucleotide sequence that extends the RNA 3′ end with a specific annealing site for a complementary fluorescent DNA oligonucleotide (Minegishi et al., 2021). By comparison with similar approaches like fluorescence-based primer extension (FPE) methodology (Ying et al., 2007), the 3′ tag was designed to form a short hairpin that minimizes the risk of hybridization to the RNA of interest before or during fluorescent-probe annealing. FluoTag-EMSA enables direct in-gel detection of sequence-specific RNA binding protein without additional processing steps, such as gel drying or blotting. This approach is compatible with quantitative assessment of RNA-protein interactions through RBP titration and binding constants determination. Because many RNA-binding domains (RBDs), including K homology (KH) domains, recognize only short single-stranded motifs, it can be challenging to reliably distinguish sequence-specific ribonucleoprotein (RNP) complexes from non-specific background binding. To address this, a built-in control in our workflow compares RNA binding of the protein of interest in the presence of increasing concentrations of unlabeled wild-type or mutant competitor RNAs. As a proof of concept, we focused on the interaction between KH domains of Bicc1 and the proximal 3′UTR of *Dand5* mRNA, which has been extensively characterized *in vitro* and validated *in vivo*, supporting the physiological relevance of the observed interactions (Minegishi et al., 2021; Rothé et al., 2025).

To further increase the versatility of this assay, we designed and validated a second fluorescent tag/probe duplex. Both duplexes can be used interchangeably with identical properties, circumventing potential off-target base pairing with the RNA of interest and enabling dual-color assays. Thus, FluoTag-EMSA supports orthogonal tagging, dual-wavelength detection, and robust quantification of dissociation constants (K_d_) and half-maximal inhibitory concentration (IC_50_), with consistent results across the two independent tag/probe pairs. The complete workflow, from transcription template to EMSA quantification, can be performed in 2 days, making this method ideally suited for both research and teaching settings.

## 2 Materials and equipment

Non-commercial buffers and solutions were prepared in-house using nuclease-free water.

For PCR amplification.

- Phire Green Master Mix 2x (Thermo Scientific™, F126L)
- PCR primer Forward and Reverse (Microsynth AG, custom design)
- Template plasmid or cDNA (obtained from in-house or commercial sources)
- Trio Thermocycler (Biometra)
- NucleoSpin PCR Clean-up (Macherey-Nagel, 740,609.50)

For *in vitro* transcription.

- SP6 transcription kit (Roche, 10 810 274 001)
- NTP mix 10 mM (Invitrogen, 18,109-017)
- RNase inhibitor (Roche, 03 335 402 001)
- DNase I (Roche, 04 716 728 001)
- Monarch® Spin RNA Cleanup Kit (NEB, T2030)

For agarose gel electrophoresis.

- AgaPure™ Agarose LE (Canvax, CANAG006)
- 10× TAE (Tris-Acetate-EDTA) buffer: 0.4 M Tris, 0.2 M acetic acid, 10 mM EDTA, pH ∼8.3
- GelRed (Biotium, 41,001)
- Gel Loading Dye Purple 6X (NEB, B7024S)
- Horizontal agarose gel electrophoresis system (VWR 700-0082)
- Standard Power Pack Power Supply (Biometra, P25)
- Bio-Rad ChemiDoc™ Imager

For EMSA.

- 10× EMSA buffer: 100 mM Tris-HCl pH 8.0, 1 M KCl, 25 mM MgCl_2_, 25% glycerol
- Fluorescent DNA oligonucleotide (probe) with 5′Dyomics 681 or Dyomics 781 fluorophore (Microsynth AG, custom design)
- Recombinant RBP (obtained from in-house or commercial sources)
- DTT (Huberlab, A1101.0005)
- Yeast tRNA (Thermo Scientific™, AM7119)
- 6× Orange G loading dye: 2 g/L Orange G (Sigma-Aldrich, 861,286), 30% glycerol

For native polyacrylamide gel preparation and electrophoresis.

- Mini-PROTEAN vertical electrophoresis cell (Bio-Rad, 1658004)
- ProtoGel (30%), 37.5:1 Acrylamide to Bisacrylamide Solution (National Diagnostics, EC8901LTR)
- 10× TBE (Tris-Borate-EDTA) buffer: 0.45 M Tris, 0.45 M Boric acid, 10 mM EDTA, pH ∼8.3
- Glycerol (Fisher Scientific, G/0650/08)
- Ammonium persulfate (APS) (Sigma-Aldrich, A3678)
- TEMED (Sigma-Aldrich, T22500-5)
- Standard Power Pack Power Supply (Biometra, P25)
- LICOR Odyssey® CLx Scanner

For image analysis.

- LICOR Odyssey® Empiria Studio
- Prism GraphPad

## 3 Methods and results

### 3.1 Rational design of 3′-Tagged RNA

**FIGURE 1:**
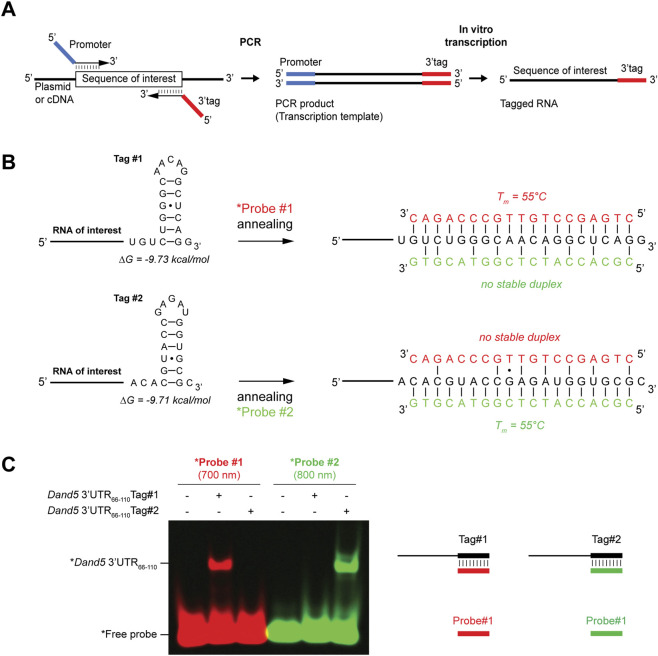
FluoTag-EMSA analysis of 3′-tagged RNAs using cognate fluorescent DNA probes. **(A)** Strategy to insert a 5′promoter sequence and a 3′tag in the transcription template using PCR. The blue and red segments correspond to the RNA polymerase promoter used for *in vitro* transcription and to the 3′ tag, respectively. **(B)** Predicted secondary structures of RNA Tags #1 and #2 alone (left) or annealed to Probes #1 or #2, respectively (right). Secondary structures and minimum free energies (ΔG) were predicted using the RNAfold server with calculations performed at 25 °C (Lorenz et al., 2011). Pairing with the non-cognate probe is also shown; it does not lead to the formation of a stable duplex. **(C)** EMSA analysis of tagged RNA-fluorescent probe duplexes performed in absence of protein according to the protocol described in the section 3.5.2.

The transcription template is generated by PCR using either a plasmid containing the sequence of interest or a cDNA pool as template. The forward primer includes the RNA polymerase promoter sequence (e.g., SP6, T7, or T3), while the reverse primer introduces the 3′tag (Figure 1A; Table 1). Both primers are bipartite: the 5′overhang (promoter or tag) does not anneal during the first PCR cycle but is incorporated in subsequent rounds. To ensure efficient initial priming, the 3′annealing portion of each primer has a melting temperature (Tm) of at least 48 °C-50 °C (calculated using the Nearest-Neighbor method). To accommodate this design, four initial PCR cycles with a reduced annealing temperature are recommended (see next section).

**TABLE 1:** Sequence and characteristics of the PCR primers and DNA probes.

RNA of interest	Variant	Tag	Primer N°	Primer sequence Fw/Rev	Length	T_a_ (°C)	T_m_ (°C)
*Dand5* 3′UTR_66-110_	WT	Tag#1	F1	**ATTTAGGTGACACTATAGAA** GAA **AGACGTGAC** CTGAATGAT	41	51	65
			R1	**C** **CTGAGCCTGTTGCCCAGAC** **A**TTCTGCTCTCTTGACCCG	39	51	72
*Dand5* 3′UTR_66-110_	WT	Tag#2	F1	**ATTTAGGTGACACTATAGAA** GAA **AGACGTGAC** CTGAATGAT	41	51	65
			R2	**G** **CGCACCATCTCGGTACGTG** **T**TTCTGCTCTCTTGACCCG	39	51	72
*Dand5* 3′UTR_66-110_	WT	-	F1	**ATTTAGGTGACACTATAGAA** GAA **AGACGTGAC** CTGAATGAT	41	51	65
			R3	TTCTGCTCTCTTGACCCGATGCAC	24	N/A	60
*Dand5* 3′UTR_66-110_	Mut	-	F2	**ATTTAGGTGACACTATAGAA** GAA **A** A **A** G **GTGAC** CTGAATGATGTGCATC	48	56	68
			R3	TTCTGCTCTCTTGACCCGATGCAC	24	N/A	60

Fluorescent probe	5′Dye	Probe sequence	Length	T_m_ (°C)
Probe #1	5′-Dyomics 681	**CTGAGCCTGTTGCCCAGAC**	19	55
Probe #2	5′-Dyomics 781	**CGCACCATCTCGGTACGTG**	19	55

Annealing (T_a_) and melting temperatures (T_m_) were calculated using the Nearest-Neighbor method. T_a_ refers to the primer region that anneals already during the first PCR cycle. Fluorescent Probe #1 and Probe #2 sequences are shown in red and green, respectively, and the Bicc1 binding motif in violet. Engineered mutations in this region are underlined. The sequence highlighted in blue corresponds to the SP6 promoter used for *in vitro* transcription of the PCR amplicon.

The RNA sequence of Tag#1 contained a stable short hairpin to minimize unintended folding or hybridization to the RNA of interest. A modified version of this sequence (Tag#2) was designed to prevent cross-hybridization to the fluorescent Probe#1 of Tag#1, but without altering the overall secondary structure, its stability, or the stability of a hybrid with Probe#2 (Figure 1B). Moreover, with the aim that both tag/probe pairs can be used interchangeably, they were designed to also share similar thermodynamic properties. As expected, thermal denaturation of 3′-tagged synthetic *Dand5* reporter RNAs and hybridization with fluorescent probes #1 or #2, respectively confirmed that the electrophoretic mobility of each probe was only shifted by transcripts containing their cognate 3′-tag, but not non-specifically by the other (Figure 1C). Our strategy of modifying the 3′-tag sequence while preserving hairpin stability can, in principle, be extended to generate a wide variety of tailor-made tag/probe duplexes depending on experimental requirements.

Tips and Rationale.

⋄ When tagging a new transcript, it is essential to confirm that the tag is structurally neutral and does not perturb RNA folding. This can be evaluated with secondary-structure prediction tools like RNAfold (Lorenz et al., 2011).
⋄ If base-pairing between the tag and the sequence of interest is predicted, the alternative version of the tag should be used. If both Tag#1 and Tag#2 are found to be unsuitable, the tag should be redesigned following the design rationale described above.

### 3.2 PCR amplification of DNA templates

DNA fragments containing the sequence of interest are amplified by PCR from a plasmid or a cDNA pool using a forward primer introducing a minimal SP6 promoter (Table 1). Alternatively, the forward primer contains a T7 or T3 promoter sequence.

Reaction mix (in 50 μL).

- 25 μL Phire Green Master Mix 2x (Thermo Scientific™)
- 1 μL primer mix (10 μM each, forward and reverse)
- 25 ng template plasmid (or cDNA)
- RNase-free water to 50 μL

Thermal cycling program.

- 1 cycle (initial denaturation):
  98 °C, 1 min.

- 4 cycles (priming the reaction):
  98 °C, 10 s.
  52 °C, 10 s.
  72 °C, 10 s.

- 30 cycles (amplification):
  98 °C, 10 s.
  62 °C, 10 s.
  72 °C, 10 s.

- 1 cycle (final extension):
  72 °C, 1 min.

**FIGURE 2:**
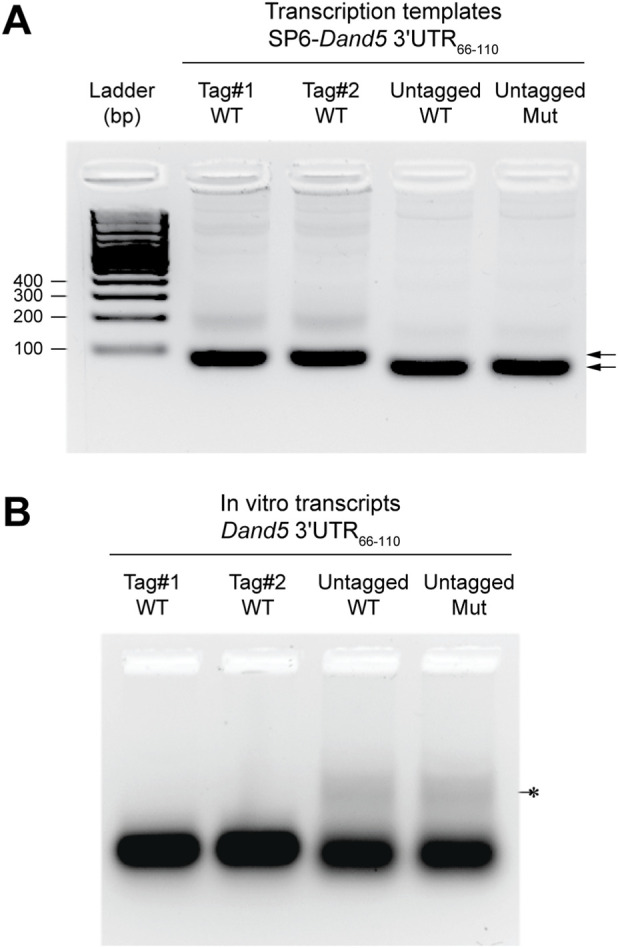
Quality control of the PCR products and their *in vitro* transcripts. **(A)** Size fractionation of purified PCR product (100 ng) on 3% agarose gel stained with GelRed. The expected sizes for tagged or untagged sequences are 86 bp or 65 bp, respectively (arrowheads). **(B)** Agarose gel electrophoresis of the indicated *in vitro* transcripts (250 ng) following column purification. A minor species of untagged RNA (<10%) migrated more slowly than the main product, likely due to alternative folding.

To PCR amplify enough DNA for subsequent *in vitro* transcription, multiple independent PCR reactions may be pooled. Purify amplicons using a DNA clean-up column (e.g., NucleoSpin PCR Clean-up, Macherey-Nagel) and elute in 30 μL RNase-free water. Quantify the DNA by measuring UV light absorbance at 260 nm and load 100 ng on a 3% agarose gel to verify the size (Figure 2A).

Tips and Rationale.

⋄ If additional bands of unexpected sizes are present, the PCR product should be isolated by gel excision before proceeding with column purification and *in vitro* transcription.

### 3.3 *In Vitro* transcription by SP6 polymerase

Reaction mix (in 50 μL).

- 5 μL 10× SP6 transcription buffer (Roche)
- 5 μL NTP mix (10 mM each)
- 1 μL RNase inhibitor (40 U/μL, Roche)
- 4 μL SP6 RNA polymerase (20 U/μL, Roche)
- 250–500 ng purified DNA template
- RNase-free water to 50 μL

Incubate at 37 °C from 2 h to overnight. Add 2 μL DNase I (10 U/μL) and incubate 1 h at 37 °C. Purify RNA using an RNA clean-up column (e.g., Monarch® Spin RNA Cleanup Kit, NEB) and elute in RNase-free water. Quantify RNA by measuring UV absorbance at 260 nm and load 250 ng on a 3% agarose gel to confirm RNA integrity (Figure 2B).

Tips and Rationale.

⋄ For short (<100 nt) or secondary structure-rich RNA, overnight transcription is recommended to maximize the yield.
⋄ Electrophoresis on native agarose gels sometimes enriches synthetic RNA in more than only a single band that are not indicative of RNA degradation, but of alternative conformations. In contrast, smeared bands suggest degradation and are problematic. Note that native agarose gels do not allow accurate size determination, which requires a denaturing gel, such as polyacrylamide-urea for short molecules (<1,000 nt) (Green and Sambrook, 2021) or agarose-glyoxal for long molecules (>1,000 nt) (Rio, 2015).
⋄ To assess the efficiency of the purification workflow, include a negative control without RNA polymerase. This control allows verification of complete DNA template removal by DNase treatment, as well as the effective elimination of DNA fragments and unincorporated nucleotides by the purification column. Additionally, it can serve as a blank for spectrophotometric quantification of RNA.

### 3.4 Native polyacrylamide gel preparation

To prepare four 5% native polyacrylamide gels using a Mini-PROTEAN–type system (10 mL per gel).

- 6.7 mL 30% acrylamide/bis solution (37.5:1)
- 2 mL 10× TBE buffer (0.45 M Tris-Borate, 10 mM EDTA)
- 2 mL 50% glycerol
- 400 μL 10% ammonium persulfate (APS)
- 20 μL TEMED
- 29 mL RNase-free water

Mix and pour the gel solution into the assembled casting plates, using 10-well or 12-well combs as needed. Pre-run the polymerized gels in 0.5× TBE with 2.5% glycerol at 70 V for 30 min to equilibrate and remove excess APS.

Tips and Rationale.

⋄ During polyacrylamide gel polymerization, TEMED catalyzes the decomposition of APS into free sulfate radicals. These radicals initiate acrylamide polymerization but can also oxidize and degrade RNA, and damage proteins, if residual APS or radicals remain in the gel.
⋄ The gels can be prepared in advance and stored for up to 24 h at 4 °C in a sealed plastic bag containing a damp tissue to prevent drying.

### 3.5 FluoTag-EMSA: RBP titration and competition assay

#### 3.5.1 Materials

- 10× EMSA buffer: 100 mM Tris-HCl pH 8.0, 1 M KCl, 25 mM MgCl_2_, 25% glycerol
- 3′-tagged RNA (2 μM)
- Unlabeled competitor RNA (4 μM)
- Fluorescent RNA probe (5 μM)
- Recombinant RBP (stock ≥10 µM)
- DTT (20 mM)
- Yeast tRNA (1 μg/μL)
- 6× Orange G loading dye

Tips and Rationale.

⋄ All reagents can be prepared in advance and stored at −20 °C for several months without loss of stability.
⋄ KCl contributes to the overall ionic strength of the reaction, dampening electrostatic repulsion between negatively charged molecules. This effect reduces nonspecific interactions and provides a favorable environment for correct RNA folding.
⋄ MgCl_2_ is essential for stabilizing RNA secondary and tertiary structures. Mg^2+^ ions neutralize the negative charges on the phosphate backbone and minimizes repulsion. They can also coordinate with defined structural motifs in the RNA to stabilize secondary and tertiary folding.
⋄ Glycerol enhances the stability of RNA and proteins during incubation, prevents aggregation, increases sample density for efficient gel loading, and can improve band resolution during electrophoresis.
⋄ Xylene cyanol and bromophenol blue are not recommended as loading dyes for assays involving far-red/near-infrared fluorescent scanning (700-800 nm) because they absorb and may emit fluorescence within this wavelength range, generating background signal that interferes with accurate detection. Instead, Orange G, which is spectrally neutral in the far-red/near-infrared region, is preferred.

#### 3.5.2 FluoTag-EMSA for K_d_ Determination

**FIGURE 3:**
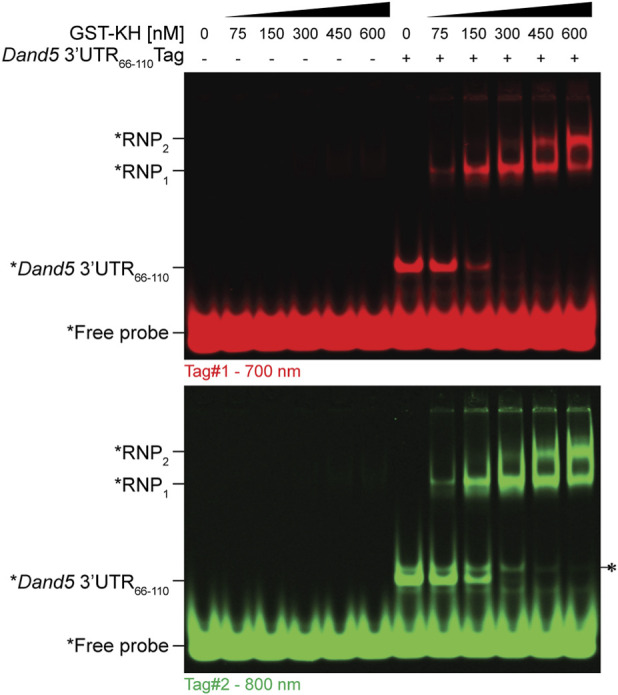
FluoTag-EMSA of GST-KH binding to *Dand5 3′UTR*_*66-110*_. As indicated, increasing concentrations of recombinant GST-KH protein were incubated with each fluorescent probe alone (control) or pre-annealed with RNA. The absence of a mobility shift with the probe alone confirms that GST-KH does not interact with the probe itself. Additional RNPs migrating with slower mobility only formed at elevated GST-KH concentrations (RNP_1_and RNP_2_), suggesting higher-order complexes consistent with the ability of the Bicc1 KH domains to oligomerize (Rothé et al., 2023). Note that a minor folding isoform of slightly lower electrophoretic mobility is visible for the Tag#2 duplex (*). This isoform undergoes a mobility shift only at high GST-KH concentrations (>300 nM), it accounts for only 10% of the total duplex population and does not influence the K_d_determination for the main isoform. The concentration-dependent formation of the fluorescent RNP in response to increasing GST-KH levels is quantified in Section 3.6 and Figure 5.

- Final reaction volume: 15 μL (See Figure 3)
- Final tagged RNA concentration: 50 nM
- Final fluorescent probe concentration: 100 nM (2-fold excess)
- Recombinant RBP titration: prepare a serial dilution of the recombinant protein in EMSA 1X buffer at 10× the desired final concentrations, e.g., to obtain final concentrations ranging from 75 to 600 nM, prepare a dilution series from 0.75 to 6 μM.

Procedure.

- Prepare a master mix excluding DTT, yeast tRNA and recombinant RBP (Table 2, Step #1). Vortex briefly.
- Denature RNA at 98 °C for 3 min, then allow to renature at room temperature in a rack for 10 min to enable annealing of the fluorescent DNA probe.
- Add DTT and yeast tRNA (Table 2, Step #2). Vortex briefly.
- Distribute 13.5 µL per tube.
- Add 1.5 µL of recombinant RBP or EMSA 1X for the control without protein (Table 2, Step #3). Gently mix.
- Incubate 30 min on ice.
- Add 3 μL 6× Orange G loading dye. Gently mix.

**TABLE 2:** Pipetting scheme for K_d_ determination.

Step	RBP final concentration [nM]*	0	75	150	300	450	600	Mix n+1
#1	H_2_O	9.975	9.975	9.975	9.975	9.975	9.975	x (n+1)
	EMSA buffer 10X	1.35	1.35	1.35	1.35	1.35	1.35	x (n+1)
	Tagged RNA 2 µM	0.375	0.375	0.375	0.375	0.375	0.375	x (n+1)
	Fluorescent probe 5 µM	0.3	0.3	0.3	0.3	0.3	0.3	x (n+1)
#2	DTT 20 mM	0.75	0.75	0.75	0.75	0.75	0.75	x (n+1)
	tRNA 1 μg/μL	0.75	0.75	0.75	0.75	0.75	0.75	x (n+1)

	RBP intermediate stock [µM]	-	0.75	1.5	3	4.5	6	
#3	EMSA 1X	1.5	-	-	-	-	-	-
	Recombinant RBP	-	1.5	1.5	1.5	1.5	1.5	-

The final concentration of recombinant RBP in a 15 µL reaction is indicated for each condition. The volumes below are indicated in µL. Importantly, DTT and tRNA must be added after the denaturation-renaturation step. Pipetting is performed as a master mix prepared for n + 1 reactions, as specified in the rightmost column. Step #3 describes the addition of recombinant RBP from intermediate concentration stocks, adjusted to achieve the indicated final concentrations. For the free RNA control condition, EMSA 1× buffer is added in place of the recombinant protein.

Tips and Rationale.

⋄ The LI-COR handbook recommends to stabilize fluorescent dyes and improve quantification accuracy by adding DTT to the master mix.
⋄ DTT is added after denaturation/renaturation steps to preserve its reducing capacity.
⋄ Yeast tRNA is commonly used as a nonspecific competitor to reduce nonspecific binding in gel shift assays.
⋄ Yeast tRNA is added to the master mix after denaturation/renaturation to prevent nonspecific annealing to the tagged RNA or fluorescent probe.
⋄ To save time, the recombinant RBP can be aliquoted into tubes during the RNA renaturation step.
⋄ To save time and minimize pipetting delays, Orange loading dye can be pre-loaded into the tube caps during incubation and briefly centrifuged into the reaction at the end of the incubation period.
⋄ To help preserve the fluorescence of far-red/near-infrared dyes, which can be diminished by prolonged light exposure, it is preferable to incubate the samples in the dark.
⋄ When implementing the assay, include a control lacking RNA to verify that the fluorescent probe alone does not bind the RBP.
⋄ Conservation of fluorescence intensity of the RNA/probe duplex (sum of free duplex and RNP band intensities) should be verified across all conditions to ensure that the protein does not perturb the heteroduplex equilibrium by displacing the probe from the RNA.

#### 3.5.3 FluoTag-EMSA competition assay

**FIGURE 4:**
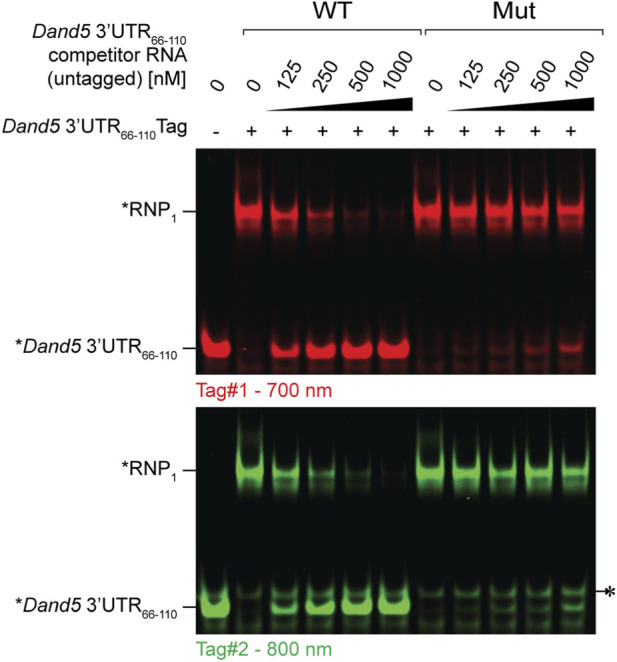
Competitive FluoTag-EMSA to assess specific binding of GST-KH to the *Dand5 3′UTR*_*66-110*_. Pre-assembled RNPs between recombinant GST-KH and the *Dand5 3′UTR*_*66-110*_were incubated with increasing concentrations of non-fluorescent competitor transcripts, as indicated. A fixed concentration of 200 nM of GST-KH was used, corresponding to ∼90% displacement of the fluorescent probe, while not affecting the mobility of the minor isoform observed with the Tag#2 duplex (*). The concentration-dependent dissociation of the fluorescent RNP in response to competitor RNA is quantified in Section 3.6 and Figure 5.

- Fluorescent RNP complexes of tagged RNA, annealed fluorescent probe, and bound recombinant RBP are assembled in a total volume of 15 μL, as described in Section 3.5.2 (See Figure 4). The stability of RNP complexes is then assessed by adding unlabeled competitor RNA, resulting in a final reaction volume of 20 µL. Note that the RBP is added at a sub-saturating fixed protein concentration yielding 75%–95% RNA binding, as determined by K_d_ measurements. Otherwise, an excess of unbound protein could unduly bias the outcome of the assay by sequestering untagged competitor RNA, reducing its availability, and altering the apparent binding equilibrium.
- Competitor RNA titration: prepare a serial dilution of the competitor RNA in EMSA 1X buffer at 4× the desired final concentrations, e.g., to achieve final concentrations ranging from 125 to 1,000 nM, prepare a dilution series from 0.5 to 4 μM.

Procedure.

- Prepare a master mix excluding DTT, yeast tRNA and protein (Table 3, Step #1). Vortex briefly.
- Denature RNA at 98 °C for 3 min, then allow to renature at room temperature in a rack for 10 min to enable annealing of the fluorescent DNA probe.
- Add DTT, yeast tRNA and recombinant protein (Table 3, Step #2). Gently mix.
- Distribute 15 µL per tube
- Incubate 30 min on ice.
- Add 5 μL of unlabeled competitor RNA or EMSA 1X for the control without competitor (Table 3, Step #3). Gently mix.
- Incubate an additional 30 min on ice.
- Add 4 μL 6× Orange G loading dye. Gently mix.

**TABLE 3:** Pipetting scheme for IC_50_ determination.

Step	Competitor final concentration [nM]*	0	125	250	500	1,000	Mix n+1
#1	H20	9.975	9.975	9.975	9.975	9.975	x (n+1)
	EMSA buffer 10X	1.350	1.350	1.350	1.350	1.350	x (n+1)
	Tagged RNA 2 µM	0.375	0.375	0.375	0.375	0.375	x (n+1)
	Fluorescent probe 5 µM	0.3	0.300	0.300	0.300	0.300	x (n+1)
#2	DTT 20 mM	0.75	0.750	0.750	0.750	0.750	x (n+1)
	tRNA 1 μg/μL	0.75	0.750	0.750	0.750	0.750	x (n+1)
	Recombinant RBP	1.5	1.5	1.5	1.5	1.5	x (n+1)

	Competitor RNA intermediate stock [µM]	-	0. 5	1	2	4	
#3	EMSA 1X	5	-	-	-	-	-
	Competitor RNA	-	5	5	5	5	-

The final concentration of recombinant RBP is fixed and determined based on the K_d_ titration described above, corresponding to the amount required to bind between 75% and 95% of the total tagged RNA. The following volumes are indicated in µL. Importantly, DTT, tRNA and recombinant protein must be added after the denaturation-renaturation step. Pipetting is performed as a master mix prepared for n + 1 reactions, as specified in the rightmost column. Step #3 corresponds to the individual addition of unlabeled competitor RNA from intermediate concentration stocks, adjusted to achieve the indicated final concentrations. For the negative control condition, EMSA 1× buffer is added in place of the competitor RNA.

Tips and Rationale.

⋄ The RBP concentration required to achieve 75%–95% RNA binding can be obtained either by graphical interpolation from the binding curve or by solving the fitted equation for x at the desired y value (see Section 3.6).
⋄ To save time and minimize pipetting delays, competitor RNA and Orange loading dye can be pre-loaded into the tube caps during incubation and briefly centrifuged into the reaction at the end of the incubation period.
⋄ To help preserve the fluorescence of far-red/near-infrared dyes, which can be diminished by prolonged light exposure, it is preferable to incubate the samples in the dark.

#### 3.5.4 Electrophoresis and imaging

- Load samples onto 5% native polyacrylamide gels (See Figures 3, Figure 4).
- Run the gels at 70 V for 1 h.
- Image the gels using a near-infrared imaging system (e.g., LICOR Odyssey® CLx Scanner) at 700 nm or 800 nm, depending on the fluorophore.

Tips and Rationale.

⋄ Before loading, rinse each well thoroughly by pipetting migration buffer up and down using a P1000 pipette to remove any glycerol accumulation.
⋄ The entirety of the sample can be loaded; however, loading only half is usually sufficient if desired for practical reasons.
⋄ To help preserve the fluorescence of far-red/near-infrared dyes, keep the electrophoresis cell in the dark, e.g., by covering it with cardboard.
⋄ Gels are typically run at room temperature; however, for labile complexes, running in a cold room (∼4 °C) or using a cooling device can be beneficial.
⋄ Fluorescence scanning can be performed directly on the gel without the need for uncasting and drying. To optimize complex separation, electrophoresis can be paused for scanning, allowing migration to resume afterward if necessary.

### 3.6 Image quantification

Using image analysis software (e.g., LI-COR Odyssey® Empiria Studio), define tightly fitted rectangles around the bands of interest (unbound and bound RNA). Some software platforms automatically calculate signal intensity using the formula Signal = Total − (Background × Area), where background subtraction corrects for non-specific fluorescence to improve quantification accuracy. If background subtraction is not automated, define a rectangle of identical size in an empty gel lane and use the measured signal as a background reference. Subtract this value from the signal intensity of all other bands to correct for non-specific background.

For each condition, calculate the percentage of bound RNA using the formula:

$$\%\text{ Bound RNA}=\left[\;{\text{Signal}}_{\text{bound}}\;/\;\left({\text{Signal}}_{\text{bound}}+{\text{Signal}}_{\text{unbound}}\right)\;\right]\times 100$$

**FIGURE 5:**
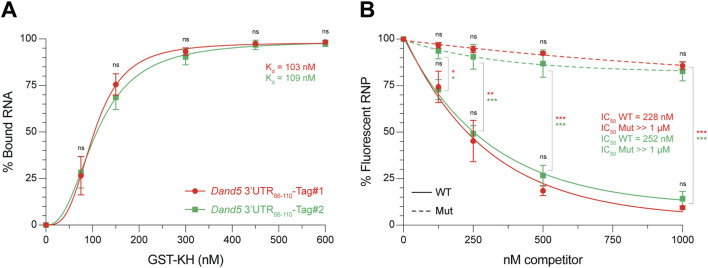
EMSA-based quantification of K_d_ and IC_50_ values of sequence-specific GST-KH binding to the *Dand5 3′UTR*_*66-110*_. **(A)** Binding curves showing the interaction between GST-KH and *Dand5* 3′UTR_66-110_ RNA labeled with fluorescent Tag#1 (in red) or Tag#2 (green) duplexes, as described in Figure 3. The percentage of bound RNA is plotted against increasing concentrations of GST-KH. Apparent dissociation constants (K_d_) are shown on the graph. They were calculated by nonlinear regression using a one-site binding model with Hill slope: Y = Bmax*X^h^/(K_d_^h^ + X^h^), where Y is the percentage of bound RNA, X is the protein concentration, Bmax is the maximal binding (plateau), K_d_ is the apparent dissociation constant, and h is the Hill slope (indicator of binding cooperativity). Data are means ± SD from 4 independent experiments. **(B)** Competition curves of pre-formed fluorescent RNPs (Tag#1 or Tag#2) by unlabeled wild-type (WT, solid lines) or mutant (Mut, dashed lines) competitor RNAs, as described in Figure 4. The percentage of fluorescent RNP is plotted against increasing concentrations of competitor RNA. IC_50_ values are shown on the graph. They were determined using a one-phase decay nonlinear regression model: Y = (Y_0_ – Plateau) × e^-kX^ + Plateau, where Y is the percentage of fluorescent RNP, X is the competitor RNA concentration, Y_0_ is the percentage of fluorescent RNP in absence of competitor, Plateau is the percentage of fluorescent RNP at the highest dose of competitor, and k is the apparent rate constant. The concentration resulting in a half-maximal decrease corresponds to X = −1/K × Ln ((50-Plateau)/(Y_0_-Plateau)). Data are means ± SD from 3 independent experiments. ns: not significant, *p < 0.05, **p < 0.01, ***p < 0.001 (Student’s t*-*test).

Plot the resulting values as a function of protein concentration for K_d_ determination or competitor RNA concentration for IC_50_ determination (Figure 5). Using graphing and statistical analysis software (e.g., GraphPad Prism), select an appropriate curve-fitting model that best fits the dataset. In practice, a coefficient of determination (*R* ^2^) ≥ 0.9 is usually considered indicative of an acceptable fit, however, since *R* ^2^ is not a reliable standalone metric for nonlinear models, additional diagnostics, including corrected Akaike Information Criterion (AICc), confidence intervals (CIs), and residuals, should also be considered (Motulsky and Christopoulos, 2004). From the fitted curve, extract the K_d_ and IC_50_ values, defined as the ligand concentration (protein, or competitor RNA, respectively) at which 50% of the fluorescent complex is formed or dissociated. No significant statistical differences were observed between the Tag#1 and Tag#2 systems in either the K_d_ determination (Figure 5A) or the competition assay (Figure 5B).

Tips and Rationale.

⋄ During fluorescence signal acquisition, measurements should be performed within the linear dynamic range. A fluorescent imager such as the Li-COR Odyssey® provides a 6-log linear range, enabling detection of a broad spectrum of signal intensities in a single scan, unlike traditional film, which is limited to ∼2 logs. Furthermore, the system automatically flags saturated pixels, preventing quantification of saturated bands.
⋄ Excess unincorporated fluorescent probe produces intense bands that may obscure the RNA/probe duplex signal. In such cases, laser power can be increased to the point of saturating the unincorporated probe band, provided the target bands remain visible and unsaturated. Display parameters (brightness and contrast) may also be adjusted to enhance visualization of target bands; on systems such as the Li-COR Odyssey®, these adjustments do not affect recorded signal values or subsequent quantification. Finally, if the free probe and the RNA/probe duplex exhibit minimal size differences, decreasing the fluorescent probe concentration to equimolarity can enhance its distinctness.
⋄ For clarity, the binding analysis presented here assumes a 1:1 protein-RNA interaction and absence of cooperativity.
⋄ The dissociation constant (K_d_) quantifies the affinity between a protein (P) and RNA (R) based on the law of mass action. The rate of complex formation is given by **v**_**on**_ **= k**_**on**_ **× [P] × [R]**, where k_on_ is the association rate constant (expressed in units of M^-1^. s^-1^). The rate of complex dissociation is **v**_**off**_ **= k**_**off**_ **× [P:R]**, where k_off_ is the dissociation rate constant (expressed in units of s^-1^) and [P:R] is the concentration of the protein-RNA complex. At equilibrium (v_on_ = v_off_), these rates are equal, so that **k**_**on**_ **× [P] × [R] = k**_**off**_ **× [P:R].** The association constant (K_a_) is expressed as **K**_**a**_ **= k**_**on**_ **/k**_**off**_ **= [P:R]/([P] × [R]).** The dissociation constant is the inverse of K_a_, so that **K**_**d**_ **= k**_**off**_ **/k**_**on**_ **= ([P] × [R])/[P:R].** K_d_ represents the concentration of ligand at which half of the complex is formed. When 50% of the RNA is bound, the concentration of free RNA [R] equals that of the complex [P:R], making their ratio [R]/[P:R] = 1. Under these conditions, K_d_ approximates the free protein concentration [P].
⋄ In EMSA, K_d_ is often approximated by the total protein concentration at which 50% of the RNA is shifted. Strictly, however, K_d_ corresponds to the free protein concentration at this point. When protein is in large excess over RNA, [P]_free_ and [P]_total_ are nearly identical, making this approximation valid. If the excess is more limited, a simple correction can be applied based on the protein-to-RNA ratio: **K**_**d**_ **= [P]**_**total**_ **– ([R]**_**total**_ **/2**) (Hulme and Trevethick, 2010). In the present case (Figure 5A), applying this correction adjusts the measured K_d_ values from 103 nM and 109 nM to 78 nM (Tag#1) and 84 nM (Tag#2), respectively.
⋄ In competition assays, a sigmoidal dose-response curve is typically observed when inhibitor concentrations span a sufficiently broad range, enabling accurate characterization of parameters such as the IC_50_. In the example shown above (Figure 5B), the limited range of competitor concentrations results in a monotonic exponential decline in signal, which is effectively captured by a one-phase decay model. While this approach does not provide mechanistic parameters, it offers a robust and reproducible method to estimate relative differences between RNA species (e.g., distinct target RNAs or sequence mutants). A more complete characterization can be achieved by expanding the concentration range of the competitor to resolve the full sigmoidal profile.
⋄ Note that apparent K_d_ and IC_50_ values obtained from EMSA are not absolute constants. They can vary with experimental conditions such as temperature, buffer composition, assay format, protein folding and purity, and the presence of cofactors or competitors.
⋄ Further guidance on binding-model choice and practical considerations for fitting and interpretation is provided in (Hulme and Trevethick, 2010; Motulsky and Christopoulos, 2004; Ryder et al., 2008).

## 4 Conclusion

FluoTag-EMSA is a practical and flexible alternative to traditional radioactive EMSA protocols. By leveraging short 3′-end RNA tags and complementary far-red/near-infrared fluorescent DNA probes, it enables safe, rapid, and highly reproducible detection of ribonucleoprotein complexes without the need for specialized equipment. The universal RNA tag that is incorporated during standard *in vitro* transcription allows modular application to any RNA of interest, eliminating the need for chemical or enzymatic labeling steps. Importantly, duplex formation does not disrupt native RNA secondary structure, as supported by biologically relevant binding profiles consistent with prior *in vivo* validation (Minegishi et al., 2021). Therefore, by combining dual-tag compatibility, orthogonal fluorescence detection, and a streamlined 2-day workflow, FluoTag-EMSA provides a novel platform for detailed analysis of protein-RNA interactions.

## 5 Limitations of the methods

Several experimental and conceptual limitations should be considered when implementing the FluoTag-EMSA protocol.

First, the fluorescent probe relies on hybridization between a structured RNA tag and a complementary DNA oligonucleotide. Because both species can form internal hairpins, a theoretical competition exists between intra- and intermolecular pairing. Although thermodynamic and kinetic calculations indicate that heteroduplex formation is strongly favored and rapidly achieved under the prescribed conditions (>99.99% duplex formation within ∼2 min), users should remain aware that custom designs deviating from the presented model should be assessed for hairpin stability (ΔG) relative to the heteroduplex in the intended buffer and at the appropriate temperature.

Second, as with any indirect labeling strategy, it is essential to verify that the protein of interest does not bind nonspecifically to the fluorescent DNA probe itself. Including probe-only controls and monitoring the conservation of total fluorescence across lanes are recommended to ensure that the protein does not displace the probe or alter the duplex equilibrium.

Finally, while the method offers flexibility through the use of two generic RNA tags, the tag sequence must be evaluated for compatibility with each RNA of interest. Computational prediction of secondary structures (e.g., with RNAfold) should be used to confirm that the tag does not interfere with the native RNA folding. If unfavorable interactions are predicted, alternative tags can be designed following the principles outlined in this work. The overall versatility of the approach thus depends on careful tag design and validation for each new target.

## Data Availability

The original contributions presented in the study are included in the article/supplementary material, further inquiries can be directed to the corresponding authors.
